# RACK1, a clue to the diagnosis of cutaneous melanomas in horses

**DOI:** 10.1186/1746-6148-8-95

**Published:** 2012-06-29

**Authors:** Cécile Campagne, Sophia Julé, Florence Bernex, Mercedes Estrada, Geneviève Aubin-Houzelstein, Jean-Jacques Panthier, Giorgia Egidy

**Affiliations:** 1 INRA, UMR955 de Génétique fonctionnelle et médicale, Ecole Nationale Vétérinaire d’Alfort, 7 avenue du Général de Gaulle, Maisons-Alfort, F-94704, France; 2 Université Paris Est, Ecole Nationale Vétérinaire d’Alfort, 7 avenue du Général de Gaulle, Maisons-Alfort, F-94704, France; 3 Ecole Nationale Vétérinaire d’Alfort, Service d’Anatomie pathologique, 7 avenue Général de Gaulle, Maisons-Alfort, F-94704, France; 4 Laboratoire IDEXX Alfort, 17 allée Jean-Baptiste Preux, Alfortville, F-94140, France; 5 Département de Biologie du Développement, Institut Pasteur, Unité de Génétique Fonctionnelle de la Souris; CNRS URA 2578, USC INRA, 25 rue du Dr. Roux, Paris, F-75724, France; 6 Present address: CRC – UMRS 872, 15 rue de l’Ecole de Médecine, Paris, F-75006, France

**Keywords:** Melanoma, Diagnosis, RACK1, MITF, PAX3

## Abstract

**Background:**

Melanocytic proliferations are common in horses but the diagnosis of malignancy is not always straightforward. To improve diagnosis and prognosis, markers of malignancy are needed. Receptor for activated C kinase 1 (RACK1) protein may be such a marker. RACK1 was originally found to characterize malignant melanocytic lesions in the Melanoblastoma-bearing Libechov minipig (MeLiM) and, later, in human patients. Our purpose was to investigate the value of RACK1 in the classification of cutaneous melanocytic proliferations in horses.

**Results:**

Using immunofluorescence, we report here that both MITF (Microphthalmia-associated transcription factor) and PAX3 (Paired box 3) allow the identification of melanocytic cells in horse skin samples. Importantly, RACK1 was detected in melanocytic lesions but not in healthy skin melanocytes. Finally, we found that RACK1 labeling can be used in horses to distinguish benign melanocytic tumors from melanomas. Indeed, RACK1 labeling appeared more informative to assess malignancy than individual histomorphological features.

**Conclusions:**

This study confirms that horses provide an interesting model for melanoma genesis studies. It establishes MITF and PAX3 as markers of horse melanocytic cells. RACK1 emerges as an important marker of malignancy which may contribute to progress in the diagnosis of melanomas in both human and veterinary medicine.

## Background

Melanocytic tumors are common in horses. Up to 18% of all skin tumors are melanocytic [1]. The true incidence may be even higher since a number of epidemiological studies do not include a histological report. Equine melanocytic tumors may occur in any body area regardless of age, sex or coat color. Most of these tumors are clinically benign at initial presentation, but two-thirds are thought to progress to malignancy and metastasize [2]. Accordingly, setting a diagnosis on histopathological analysis alone can be challenging [2]. The term “melanoma” is used for malignant melanocytic tumors, whereas “melanocytoma” refers to the benign forms, with the corresponding restrictions. Indeed morphological criteria are not always predictive of clinical features [1]. In human melanoma, morphology-based melanoma classification has presented limited clinical relevance. Nevertheless, Bastian and colleagues have recently shown that clinical and morphologic features associated with known mutations could be used to identify biologically related disease groups [3,4]. Coat color genetic studies in horses identified genes responsible for associated pathologies [5,6], like melanoma in gray horses [7]. More studies are needed to determine the mutation status of horse melanocytic proliferations.

At present, a molecular marker of malignancy would be of great interest to distinguish benign from malignant melanocytic tumors. We found that immunodetection of RACK1 (Receptor for activated C kinase 1) protein deserves consideration as such a marker. Indeed RACK1 is strongly detected in melanoma cells of primary tumors and metastases developed in MeLiM minipigs as well as in human patients. In contrast, RACK1 is not detected in normal skin melanocytes or in benign tumoral proliferations [8].

RACK1 is a 36 kDa scaffold protein containing seven internal WD40 repeats, originally identified as an anchoring protein for protein kinase C (PKC) [9]. It is now well established that RACK1 is ubiquitous, with a tightly regulated expression, and that it interacts with a large number of proteins. Through its ability to coordinate the interaction of key signaling molecules, RACK1 is widely perceived as playing a central role in critical biological responses both in normal cell physiology and in tumorigenesis [10]. Several *in vitro* studies have shown that RACK1 could be implicated in cancer hallmarks [10-14]. Particularly, there is evidence for a role of RACK1 in the pathogenesis of melanoma. In the MeWo human melanoma cell line, RACK1 serves as an adaptor protein for PKC-mediated JNK (c-Jun NH2-terminal kinase) activation and increases the survival to UV induced-apoptosis [15]. RACK1 may allow cross-talks between several pathways involved in melanoma development through the orchestration of protein-protein interactions.

In this study, we tested the value of RACK1 detection in the diagnosis of horse melanoma.

## Methods

### Horse (Equus ferus caballus) tissues

Horse tissues were submitted as formalin-fixed excisional biopsies to the Alfort Veterinary Medicine School (*n =* 12), and at the IDEXX Diagnosis Laboratory (*n =* 27). Skin samples consisted in previously diagnosed cutaneous melanomas (*n =* 9) or melanocytomas (*n =* 15) (Table 1), and in normal glabrous skin (*n =* 6) from under the tail area and lips, as well as normal furry skin from the rump (*n =* 6), used as control. Tissues that were not archival diagnostic material were taken at the programmed euthanasia of individuals used for another study. Euthanasia had been practiced following recommended consensus publications. Tumors were selected such that cytological characteristics or vascular emboli indicated a clear diagnosis. Briefly, dermal or epidermal proliferations of nests of melanocytes with variable degrees of pigmentation were considered melanocytomas when they were well delimited, without or with low atypia, no mitoses or less than 2 mitoses per ten high power fields, and no signs of vascular invasion or vascular embolus. Melanomas were characterized by anisocytosis, anisokaryosis, prominent nucleoli, nuclear atypia or alternatively tumoral vascular emboli. A high mitotic index corresponding to more than 10 mitoses per ten high power fields was also considered a mark of malignancy, however samples with low or moderate mitotic index could carry other characteristics of malignancy.

**Table 1:** Epidemiological data from horses with the melanocytic lesions examined

**Samples**	**Age**	**Sex**	**Breed**	**Localisation**	**Mitoses**
Melanocytoma 1	16	G	-	trunk	None
Melanocytoma 2	-	M	-	trunk	None
Melanocytoma 3	5	G	Lusitanian	under tail	None
Melanocytoma 4	-	F	-	trunk	None
Melanocytoma 5	10	F	-	trunk	Low
Melanocytoma 6	-	M	Connemara	limb	Low
Melanocytoma 7	16	F	Connemara	trunk	Low
Melanocytoma 8	5	F	-	limb	Moderate
Melanocytoma 9	11	M	-	trunk	High
Melanocytoma 10	3	G	French Saddlebred	head	Low
Melanocytoma 11	-	F	-	trunk	Low
Melanocytoma 12	5	G	Appaloosa	trunk	Low
Melanocytoma 13	-	F	-	trunk	None
Melanocytoma 14	7	F	French Saddlebred	head	Low
Melanocytoma 15	12	-	-	limb	Low
Melanoma 1	25	-	French Saddlebred	under tail	High
Melanoma 2	2	G	-	trunk	High
Melanoma 3	14	M	French Saddlebred	foreskin	Low
Melanoma 4	17	M	Highland	under tail	High
Melanoma 5	-	M	-	trunk	Moderate
Melanoma 6	-	M	-	under tail	Moderate
Melanoma 7	11	G	French Saddlebred	limb	Low
Melanoma 8	3	G	-	trunk	Low
Melanoma 9	4	F	Anglo-arabian	under tail	Low

M: Male; F: Female, G: Gelded, -: unavailable data.

Age of animals, when available, ranged from 2 to 25 years. In our sampling aged gray horses have a low representation (Table 1) despite the high incidence of slow evolving melanocytic tumors in these horses [16,17].

### Immunostaining and ApoTome microscopy

The protocol for immunofluorescence was as previously described [8] with minor modifications. Briefly, antigen retrieval was performed in Tris-EDTA, pH 9, for 30 min in a water-bath. Antibodies were mouse monoclonals anti-MITF (Zymed, dilution 1:50; Invitrogen, Cergy-Pontoise, France) anti-RACK1 (Transduction Laboratories, 1:150; BD Biosciences, Le Pont de Claix, France) and rabbit polyclonals anti- cytokeratin5 (Covance; 1:1000; Eurogentec, Angers, France) and anti-PAX3 (Zymed; 1:200). Nuclear counter-staining was achieved with 4', 6'-diamidino-2-phénylindole (DAPI) (Invitrogen, 1:1000). Sections were examined with a Zeiss Axio Observer Z1M ApoTome microscope (Carl Zeiss S.A.S. ; Le Pecq, France). Controls without the first antibodies showed no unspecific labeling. Images were processed with the *AxioVision* computer program version 4.6 (Carl Zeiss). Figures are representative of the skin samples evaluated. All images shown are individual sections of z series stack. Final figures were assembled with Adobe Photoshop CS3 (Adobe Systems; USA).

### Analysis of RACK1 staining distribution

RACK1 staining distribution was analyzed at the tissular and cellular levels. Distribution within the cytoplasm was graded 0 when homogeneous and 1 when heterogeneous. Samples were graded blindly without reference to pathology reports.

### Analysis of elementary histological features

All histopathological evaluations were carried out on routinely stained hematoxylin-eosin-safran sections. The size of the tumors was missing in some clinical files; the dimensions of the lesions on histological sections ranged from 4 to 80 mm. To highlight the histological specificity of horse melanomas and melanocytomas, all tissue sections were examined at 3 different magnifications (10, 20, 40 high power field) and classified according to the eleven histo-morphometric criteria previously defined by Viros et al. [4]: scatter of intraepidermal melanocytes, nest formation of intraepidermal melanocytes, cytoplasmic pigmentation of neoplastic melanocytes, size and shape of cells, nuclei and nucleoli, epidermal contour, lateral circumscription, thickness of normal epidermis and presence of ulceration. Grading was carried out like in Viros et al. [4] except for ulceration which was graded 0 for absence and 1 for presence. The samples were graded blindly independently by two of us without reference to pathology reports, Two groups –melanomas and melanocytomas– were subsequently made based on these reports. Sections were observed with a Leica DMLB microscope (Leica Microsystems S.A.S., Nanterre, France). Images were processed with the *MetaVue Imaging System* (Molecular Devices; St Grégoire, France) computer program. Histological pictures were taken with AxioImager.ZI through a AxioCam HRc camera and processed with the AxioVision 4.6.3 SPI software (Carl Zeiss).

### Statistical analysis

Statistical differences between means taken in pairs were evaluated by Student’s *t* test. The test was adapted for a number of samples below 30 [18]. A *P*-value <0.05 was considered as statistically significant.

## Results

### MITF is a sensitive marker to identify melanocytic cells in horses

**Figure 1:**
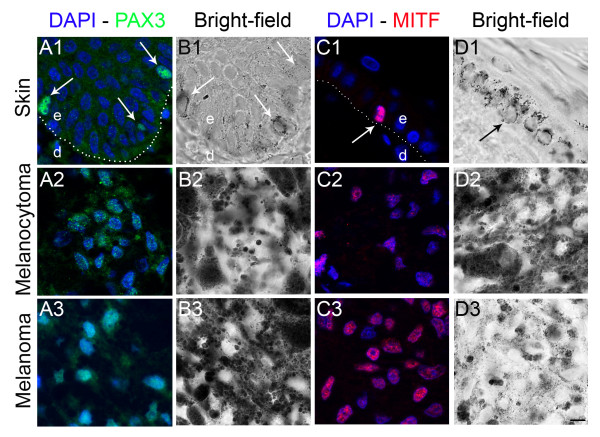
** PAX3 and MITF immunolabeling in horse skin and cutaneous melanocytic proliferations.** (**1**) control horse skin, (**2**) cutaneous melanocytoma, (**3**) cutaneous melanoma (**A**): PAX3 protein immunolabeling (green) with corresponding bright-field photographs (**B**). A specific nuclear PAX3 labeling is identified in melanocytes (arrows) and melanocytic cells (A1-A3) with low background signal. (**C**): MITF protein immunolabeling (red) with corresponding bright-field photographs (**D**). A specific nuclear MITF labeling is observed in melanocytic cells in control skin and lesions (C1-C3) with very low background. Nuclear counterstaining is shown in blue. Dotted lines (A1 and C1) indicate epidermis-dermis boundary. e, epidermis; d, dermis. Magnification is the same in all images, bar: 10 μm.

In order to analyze melanocytic proliferations, we first needed a marker to identify melanocytic cells within tissue sections. Both MITF and PAX3 transcription factors are expressed by melanocytes and their precursors [19,20]. Comparison of horse and human protein sequences for MITF and PAX3 resulted in more than 90% identity. On tissue sections, melanocytes at the basal layer of healthy skin were labeled by a specific nuclear signal using a rabbit PAX3 antibody (Figure 1 A1) or a mouse MITF antibody (Figure 1 C1). Unspecific labeling was not detected (not shown). Moreover, MITF and PAX3 are expressed by melanocytic cells within tumoral proliferations [8,21]. PAX3 and MITF-positive cells were identified both in melanocytomas and in melanomas regardless of the pigmentation of the lesion (Figure 1 A2, A3, C2, C3). Both MITF and PAX3 antibodies proved to be helpful in identifying the melanocytic lineage in horse tissues. Nevertheless, MITF antibody detected the nucleus of melanocytic cells with more sensitivity than did the PAX3 antibody. MITF antibody was thus used for further analyses.

### RACK1 protein distinguishes melanoma from melanocytoma, but also from normal melanocytes in horses

**Figure 2:**
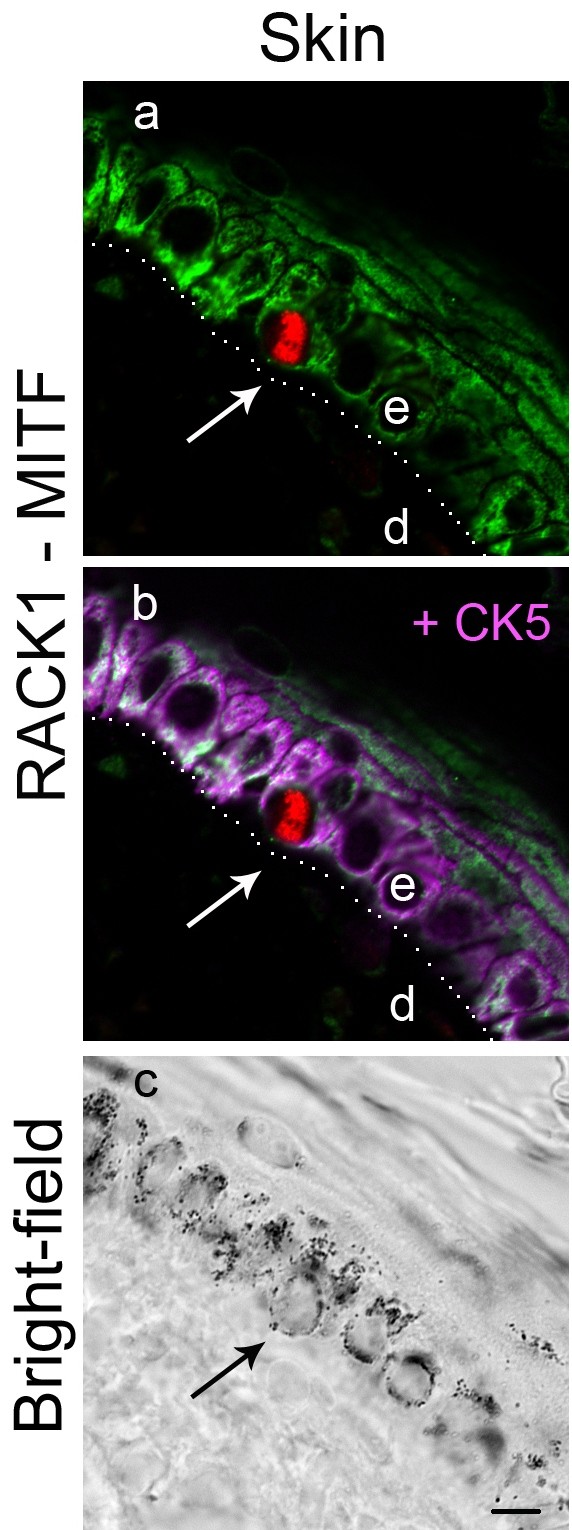
** RACK1, CK5 and MITF immunolabelings in horse skin.** RACK1 protein labeling (green), MITF (red) and CK5 (magenta) in control horse skin. Cytoplasm of basal keratinocytes is positive for CK5 signal and RACK1 (B). Melanocytes are positive for MITF, but negative for CK5 and RACK1 (A, B). Dotted line indicates epidermis-dermis boundary. e, epidermis; d, dermis. Bar: 10 μm.

We analyzed the tissue and cellular distribution of RACK1 protein, in healthy skin and melanocytic lesions, after double immunostaining for RACK1 and MITF. In healthy control skins (*n =* 12), RACK1 protein was highly expressed in the cytoplasm of keratinocytes which were used as positive controls (Figure 2A). By contrast, MITF-positive melanocytes were negative for RACK1 (Figure 2A). Triple immunostaining against RACK1, MITF and cytokeratin 5 (CK5), a marker of basal keratinocytes, was performed in order to better identify the cell type containing RACK1 in the epidermis. Every signal for RACK1 in the vicinity of melanocytes colocalized with CK5 (Figure 2B). Thus, in these labeling conditions, RACK1 was not detected in horse normal melanocytes.

**Figure 3:**
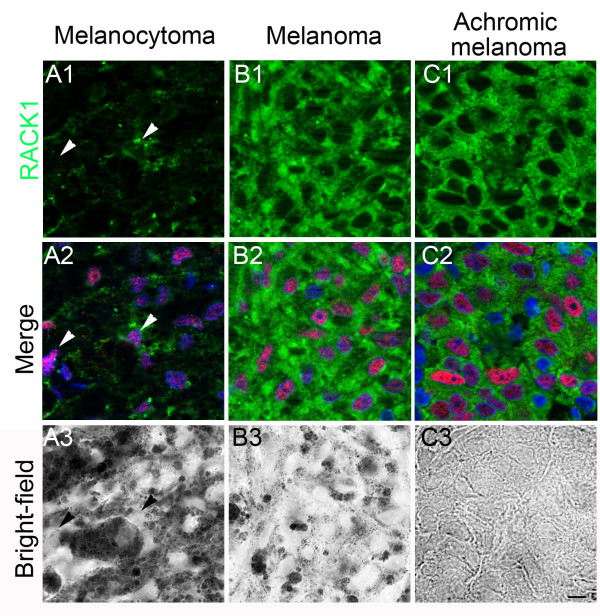
** RACK1 and MITF immunolabelings in cutaneous melanocytic proliferations from horses.** MITF labeling is shown in red and RACK1 in green. (**A**) melanocytoma, (**B**) melanoma, (**C**) achromic melanoma. In melanocytoma, RACK1 cytoplasmic expression is heterogeneous (A1 and A2). Arrowheads point to melanocytic cells that express variable amounts of RACK1. By contrast, in pigmented or achromic melanoma (B1 to C2), all MITF-positive cells display a strong and homogeneous cytoplasmic RACK1 signal (B2, C2). Nuclear counterstaining is shown in blue. Corresponding bright-field photographs are presented (A3 to C3). Magnification is the same in all images, scale bar represents 10 μm.

In all melanocytic lesions examined (*n =* 24), RACK1 was extensively detected in MITF-positive cells, with two distinct distribution patterns. RACK1 was distributed either heterogeneously (Figure 3A) or homogeneously over the lesion, whether tumors were pigmented or not (Figure 3B, 3C). In lesions with heterogeneous distribution, RACK1 was detected as a granular cytoplasmic staining in melanocytic cells. In sharp contrast, in lesions with homogeneous distribution, RACK1 staining was diffuse, perinuclear and cytoplasmic (compare Figure 3A with Figure 3B and 3C). We tested whether the cytoplasmic distribution of RACK1 staining could be of help in classifying melanoma lesions. For this purpose the distribution of RACK1 was graded 0 when homogenous, and 1 when heterogeneous. Samples were graded independently by two of us, blindly without reference to pathology reports. Subsequently, melanocytoma and melanoma samples were grouped based on pathology reports. Comparison of RACK1 grading between the two groups resulted in a statistical difference (1 ± 0 vs. 0.2 ± 0.36 respectively; *P* < 0.001). All samples histologically classified as melanocytomas (*n =* 15) stained heterogeneously for RACK1. Among melanomas (*n =* 9), two samples also stained heterogeneously for RACK1 (data not shown). Noteworthy, these two melanomas had several characteristics of low histopathological aggressiveness, which was low mitotic rate and no vascular embolisation but high anisokaryosis. All other melanoma samples stained homogeneously for RACK1. Thus, cytoplasmic RACK1 labeling may be helpful in distinguishing melanoma from benign melanocytic skin tumors.

### RACK1 detection is more informative than individual histomorphological features

**Figure 4:**
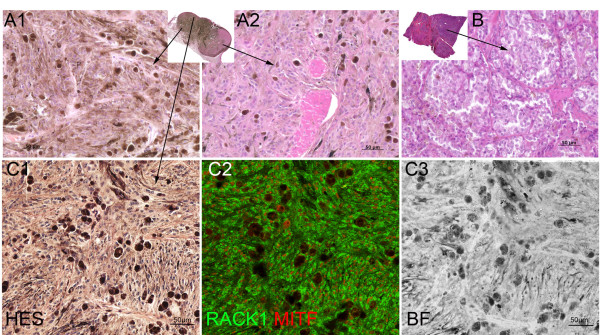
** Variability of histological features of horse melanomas and uniformity of RACK1/MITF labeling.** (**A**-**B**): Sections of two different melanomas with hematoxylin-eosin-safran staining, with their respective low magnification in the inserts. Note the variability in pigmentation between tumors (**A, B**) and in different areas of the same tumor (A1, A2). (**C**): Histological staining of a pigmented area from A with ovoid and spindled cells (C1), and low power capture of RACK1 (green) - MITF (red) labeling in the adjacent section (C2), (C3) bright-field corresponding to C2. Note RACK1 signal uniformity in the different areas. Bar: 50 μm.

Melanocytic lesions in our samples displayed a large histological variety. Figures 4A and 4B show various pigmentation patterns and cell shapes in two representative melanomas. Interestingly enough, RACK1 distribution pattern was constant throughout the whole of the lesions, as illustrated in Figure 4C. To better characterize the lesions, we performed a detailed analysis based on the morphological criteria Bastian’s group used on human samples [4], summarized in Table 2. We found that equine melanomas were characterized by: high pigmentation (Table 2, 2^nd^ column), abrupt lateral circumscription (Table 2, 4^th^ column) at the transition from involved area to adjacent normal tissue (Figure 4A) and a thicker epidermal contour (Table 2, 5^th^ column), indicative of epidermal hyperplasia. Moreover, we often observed an absence of junctional component, as previously described [17]. However none of these characteristics reached statistical significance when compared to those of melanocytomas. Finally, we checked every morphological criterion and the RACK1 distribution pattern against the malignancy status. RACK1 signal pattern of distribution was more frequently indicative of malignancy than any single morphological criterion, pointing out its usefulness as a diagnostic marker.

**Table 2:** Histomorphological features and RACK1 pattern in horse melanocytic lesions

	**Scatter**^**a**^	**Pigment**^**b**^	**Nesting**^**c**^	**Circum**^**d**^	**Epid. Contour**^**e**^	**Cell size**^**f**^	**Cell shape**^**g**^	**Nuclear size**^**h**^	**Nuclear shape**^**i**^	**Nucleolar size**^**j**^	**Ulcer**^**k**^	**RACK1 pattern**^**l**^
**Melanocytomas**												
**1**	3	4	3	-	-	3	0.5	2	-	-	0	1
**2**	-	4	0	-	-	2	1	1	-	1	-	1
**3**	0	1	2	2	2	2	2	2	1	1	0	1
**4**	1	3	3	1	4	1.5	3	3	1.5	1	1	1
**5**	1	3.5	0	2	2	2	1	1	0	1	0	1
**6**	3	4	1	1	2	3	0.5	2	0	1	1	1
**7**	1	2	2	0	2	3	0	3	0	1	0	1
**8**	0	1	1	2	2	3	1.5	2.5	1.5	3	0	1
**9**	0	3	1	0	2	3	1	2	0	1	0	1
**10**	2	3	1	1	2	2	1.5	3	0	1	0	1
**11**	1	4	0	1	3	2.5	2.5	1	1	1	0	1
**12**	0	3.5	0	0	2	3	1.5	2	0	1	1	1
**13**	2	4	0	2	3	1	2	3	0	1	0	1
**14**	1	4	2.5	0	2	2.5	1.5	2	0	1.5	0	1
**15**	-	4	0	1	0	2	1.5	2	0	1	1	1
**Melanomas**												
**1**	3	2	3	1	-	3	1	3	-	3	1	0
**2**	3	4	2	2	3	3	2.5	3	0	1	0	0
**3**	2	1	1	2	4	2	2.5	3	2	1	1	0
**4**	-	1	0	1	-	3	1	3	0	3	0	0
**5**	3	4	1	1	1	3	1	3	0	2.5	1	0
**6**	1	4	1	2	3	2	2	3	1.5	2	0	0
**7**	1	4	3	2	2	3	1	2.5	-	2	0	1
**8**	0	3	0	2	2	3	1	3	0	2.5	0	1
**9**	2	3	2	1	3	3	3	3	1	3	0	0

^a^Scatter of intraepidermal melanocytes: 0, absent; 1, slight; 2, medium; 3, prominent.

^b^Cytoplasmic pigmentation of neoplastic melanocytes: 0,absent; 1, slight; 2, medium; 3, high; 4, very high.

^c^Nesting of intraepidermal melanocytes: 0, absent; 1, slight; 2, medium; 3, prominent.

^d^Lateral circumscription: 0, discontinuous; 1, continuous; 2, abrupt.

^e^Epidermal contour: 0, atrophy; 1, effacement; 2, normal; 3, thickening; 4, hyperplasia.

^f^Cell size: 1, small; 2, medium; 3, large.

^g^Cell shape: 0, round; 1, ovoid; 2, elongated; 3, spindled.

^h^Nuclear size: 1, small; 2, medium; 3, large.

^i^Nuclear shape: 0, round; 1, ovoid; 2, elongated; 3, spindled.

^j^Nucleolar size: 1, small; 2, medium; 3, large.

^k^Ulceration: 0, absent; 1, present.

^l^RACK1 distribution pattern: 0, homogenous; 1, heterogenous.

-: Impossible evaluation.

x.5: means of two different authors observations.

## Discussion

Early identification of malignant melanocytic lesions is crucial for patient survival in human and veterinary medicine. RACK1 is a scaffold protein found to integrate various metabolic pathways involved in tumorigenesis [22,23]. It was proposed as a marker of malignancy in pig and human melanomas [8]. We extend these observations to melanomas in horses which displayed an overexpression of RACK1 when compared to normal cutaneous melanocytes.

In an attempt to find a more refined morphological classification to distinguish benign from malignant lesions, we used the criteria defined for human melanoma in equine melanocytomas and melanomas. Although none of the morphological criteria taken separately were powerful enough to distinguish between benign and malignant tissue, we highlight specific histological features of equine melanoma that recapitulate those seen in atypical rare forms of human malignant nævi and melanomas such as pigment synthesizing melanoma, desmoplastic melanoma, primary dermal melanoma, and malignant blue nævus [24-27]. This makes the study of equine melanoma a source of information to understand development of atypical melanoma in mammals.

We show that PAX3 and MITF immunolabeling can be used to identify melanocytes as well as melanocytic transformed cells within a tumor bulk. MITF is known to mark melanocytic proliferations in humans and pigs [8]. More specifically, we show that PAX3 is expressed by mature melanocytes in horses, as in humans [21]. In every sample from healthy and tumoral tissues, the melanocytic cells displayed a specific nuclear MITF labeling. Achromic tumoral samples were also positively stained for MITF. We therefore propose MITF immunolabeling as a diagnosis marker for melanocytic tumors in horses.

All the samples identified as melanocytomas by histopathologic analysis stained heterogeneously for RACK1. On the other hand, a homogeneous staining for RACK1 only appeared on melanomas. Two samples identified as melanomas also stained heterogeneously for RACK1. Both had morphological characteristics of low aggressiveness as a low mitotic index. This is a common pitfall in histological analysis of equine melanoma, and reports on antigens related to the cell cycle gave controversial results [17,28]. However, no classification based on detailed staging [29] is available for equine melanomas. We extended previous observations on RACK1 expression in melanoma from humans and pigs to melanocytic lesions in horses. We show here that the cellular distribution of RACK1 reflects melanoma progression and aggressiveness: the more homogenous and diffuse the cytoplasmic RACK1 labeling is, the more aggressive the melanoma. Thus, we propose RACK1 as a marker of malignancy in equine melanocytic proliferations.

Furthermore, our data show that the cellular distribution pattern of RACK1 on melanocytes in tumors, i.e. homogenous if malignant and heterogeneous if benign, is constant throughout the whole lesion. RACK1 distribution pattern analyzed in a punch biopsy appears as informative for diagnosis and less invasive than a complete biopsy. This is a particularly interesting element since skin biopsies in horses are not that easy to carry out. A quick grading could influence on the decision of treating melanomas more urgently, even if they are less suited for surgical therapies.

In domestic animals, veterinary pathologists use the term melanocytomas to describe benign melanocytic tumors [30]. In humans, such benign melanocytic tumors are designated as nævi and the term melanocytoma is seldom used. It defines melanocytic tumors with uncertain malignancy status [31,32]. Our data show that the distribution of RACK1 labeling is heterogeneous in horse melanocytomas while it is absent or faint in human nævi [8]. This confirms the difference between melanocytomas and nævi at the molecular level. In horses, the second most common clinical presentation of melanoma is the malignant transformation of a melanocytoma [33]. Based on RACK1 distribution, we hypothesize that horse melanocytomas may be premalignant entities ready to switch to malignancy. RACK1 detection would reveal this switch to malignancy, if any.

## Conclusions

RACK1 protein was detected with intense, diffuse and homogenous staining in MITF-positive cells of equine melanomas. This observation is consistent with stainings in human melanomas and MeLiM minipig melanocytic tumors. We now confirm the usefulness of RACK1 labeling as a diagnosis marker for melanoma. The homogenous distribution pattern of RACK1 signal shared by human, pig, and horse melanomas strongly suggests a function for RACK1 in melanoma progression in mammalian skin.

## Abbreviations

CK5: Cytokeratin 5; IGF1R: Insulin-like growth factor-1 receptor; JNK: c-Jun NH_2_-terminal kinase; MITF: Microphtalmia-associated transcription factor; Pax3: Paired box 3 gene; RACK1: Receptor for activated C-kinase 1.

## Competing interests

The authors declare that they have no competing interests.

## Authors' contributions

CC carried out immunolabelling experiments and analysis, histo-morphological analysis, statistical analysis and drafted the manuscript. SJ carried out microtome sections of samples, HES staining and immunolabelling experiments. FB participated to histo-morphological analysis. ME provided and selected samples. FB, GAH and JJP provided intellectual input and revised the manuscript. GE designed and supervised the overall study, performed control skin biopsies and drafted the manuscript. All authors read and approved the final manuscript.
